# Synthetic Biology Outside the Cell: Linking Computational Tools to Cell-Free Systems

**DOI:** 10.3389/fbioe.2014.00066

**Published:** 2014-12-09

**Authors:** Daniel D. Lewis, Fernando D. Villarreal, Fan Wu, Cheemeng Tan

**Affiliations:** ^1^Integrative Genetics and Genomics, University of California Davis, Davis, CA, USA; ^2^Department of Biomedical Engineering, University of California Davis, Davis, CA, USA

**Keywords:** synthetic biology, *in vitro* model, cell-free systems, artificial cells, computational modeling, predictive modeling, deterministic and stochastic simulations

## Abstract

As mathematical models become more commonly integrated into the study of biology, a common language for describing biological processes is manifesting. Many tools have emerged for the simulation of *in vivo* synthetic biological systems, with only a few examples of prominent work done on predicting the dynamics of cell-free synthetic systems. At the same time, experimental biologists have begun to study dynamics of *in vitro* systems encapsulated by amphiphilic molecules, opening the door for the development of a new generation of biomimetic systems. In this review, we explore both *in vivo* and *in vitro* models of biochemical networks with a special focus on tools that could be applied to the construction of cell-free expression systems. We believe that quantitative studies of complex cellular mechanisms and pathways in synthetic systems can yield important insights into what makes cells different from conventional chemical systems.

## Introduction

Synthetic biologists seek to simplify the understanding of biological systems by constructing biochemical pathways and building computational models to simulate the behavior of those pathways (De Jong, 2002; Andrianantoandro et al., 2006). This shift toward an engineering model of experimentation has revealed intriguing details about the architecture of biological networks (Milo et al., 2002), but has largely neglected to integrate older methods of biological inquiry, especially *in vitro* biology.

*In vitro* synthetic biology is an emerging area that focuses on complex biosynthesis, directed evolution, and reconstitution of biological functions (Forster and Church, 2007; Hodgman and Jewett, 2012; Swartz, 2012; Guterl and Sieber, 2013). *In vitro* reactions, also termed cell-free systems in this review, are defined as a collection of biochemical components used to quantify properties of biological systems and/or produce biological products, such as nucleic acids, polypeptides, or metabolites. Conventional *in vitro* systems are routinely used in biochemistry to measure binding affinity (Shutt and Cox, 1972; Poland et al., 1976; Strauch, 1995), assess reactivity (Waugh, 1954; Bekhor et al., 1969; Assmann and Brewer, 1974; Mari, 2002), and determine molecular structure (Solomon and Varshavsky, 1985; Weeks, 2010) of cellular components. Reconstituted *in vitro* systems are used to demonstrate the molecular basis of transcription and translation *in vivo* (Hoagland et al., 1958; Nathans et al., 1962). *In vitro* systems are also used in high-throughput screening of proteins (Hanes and Pluckthun, 1997; Zhu et al., 2001; Goshima et al., 2008), RNA (Koizumi et al., 1999; Robertson and Ellington, 1999; Goler et al., 2014), and DNA (Higuchi et al., 1988). High-throughput screening of RNA compounds is often used in directed evolution experiments to develop riboswitches and other auto-catalytic RNA structures that have utility in biosynthetic applications (Koizumi et al., 1999; Robertson and Ellington, 1999; Goler et al., 2014). In these examples, *in vitro* systems are applied as minimal and biomimetic model systems to study single cellular components in isolation (Zubay, 1973). These properties of *in vitro* systems resonate with the bottom-up approaches of synthetic biology and have indeed been exploited to create complex circuitry in cell-free systems (Kim et al., 2006; Kim and Winfree, 2011; Padirac et al., 2012). For example, an *in vitro* oscillator was developed by using cellular machinery to transcribe a pair of nicked-promoter constructs (Kim and Winfree, 2011). The first construct produces a transcript that inhibits the second construct by strand displacement, while the second construct produces an RNA oligo that activates the first construct (Kim and Winfree, 2011). As a result, the system forms a negative feedback loop that produces oscillation in the activities of the promoters (Kim and Winfree, 2011).

*In vitro* systems can be integrated with other materials to create hybrid constructs (Holtz and Asher, 1997; Murakami and Maeda, 2005; He et al., 2012; Langecker et al., 2012; Singh et al., 2013; Munkhjargal et al., 2014). A recent work develops freeze-dried *in vitro* reactions stored on paper disks (Pardee et al., 2014). These *in vitro* systems are easily stored and can be activated with water, greatly increasing the portability and flexibility of cell-free systems for applications in mobile diagnostic systems. Another intriguing class of hybrid constructs is the encapsulated cell-free system, which can also be referred to as artificial cells. These artificial cells were originally created to study the origin of cellular life, but have recently been used as biomimetic systems to address other biological questions. Pioneer work on these systems include the synthesis of poly-A RNA in self-reproducing vesicles (Walde et al., 1994), the replication of an RNA template in liposomes (Oberholzer et al., 1995b), and the compartmentalization of PCR (Oberholzer et al., 1995a). These work demonstrated that enzymatic activity could occur inside a liposome and direct the *de novo* synthesis and replication of nucleic acids. Further efforts in the field yielded enzymatic synthesis of membrane lipids inside liposomes to increase compartment size (Wick and Luisi, 1996), evidence of base pair recognition between components of a phosphatidyl nucleoside membrane (Berti et al., 1998), and poly(Phe) production inside liposomes loaded with ribosomal components (Oberholzer et al., 1999). With the advent of encapsulated protein synthesis, there was a focused attempt to reproduce key features of cellular systems using artificial cells, including the production of functional proteins (Yu et al., 2001), implementation of a transcriptional cascade (Ishikawa et al., 2004), and the membrane targeting of a translated protein (Noireaux et al., 2005).

In addition, artificial cells can perform fundamental functions associated with natural cells, such as formation of membrane pores via alpha hemolysin expression (Chalmeau et al., 2011), execution of genetic programs like a positive feedback loop (Kobori et al., 2013), and other processes associated with sensing and responding to the environment (Martini and Mansy, 2011; Lentini et al., 2014). While artificial cells cannot currently undergo self-reproduction (Noireaux et al., 2011), they have been used to gain insight into features of natural cells, including molecular crowding (Sokolova et al., 2013; Tan et al., 2013), compartmentalization (Matsuura et al., 2012), and RNA-facilitated encapsulation (Black et al., 2013). Furthermore, artificial cells have potential applications in drug delivery (Safra et al., 2000; Kaneda et al., 2009), biosensors (Martini and Mansy, 2011; Hamada et al., 2014; Lentini et al., 2014), biosynthesis (Kuruma et al., 2009; Moritani et al., 2010; Maeda et al., 2012), and bioenergy. Due to the tractability and bio-compatibility of artificial cells, they represent a potentially safer strategy when compared to natural cells for targeted therapeutic treatment.

Perhaps, the most intriguing ramification of developing *in vitro* synthetic biology will come from the establishment of new algorithms to simulate the behavior of these systems. Computational models of artificial cells could unite chemical and biological theory, combining the defined and predictable nature of *in vitro* reactions with the robust and sensitive qualities of natural cells. To date, however, computational tools for modeling artificial cell systems have not been established.

The computational toolbox for cell-free synthetic biology could be developed using two sources of models. On the one hand, physical models of single cellular components can be created from first principles, which lead to conventional focus on tools to predict structure and dynamics of single components (Bradley et al., 2005; Park et al., 2006; Tang et al., 2006; Zanghellini et al., 2006; Shaw et al., 2010; Lindorff-Larsen et al., 2011; Wijma and Janssen, 2013). For example, the enzyme glycoxylase II was re-designed to lose its original catalytic action and instead carry a functional beta-lactamase domain, which conferred antibiotic resistance to bacteria that carried the modified protein (Park et al., 2006). On the other hand, the advent of systems biology creates a wide-range of mathematical models for predicting system dynamics of natural cells (Klumpp et al., 2009; Jamshidi and Palsson, 2010; Park et al., 2010; Scott et al., 2010). Computational tools have been used to describe diverse biological functions, including somitogenesis, T-cell antigen discrimination, and heterogeneous vesicle formation (Lewis, 2003; Altan-Bonnet and Germain, 2005; Heinrich and Rapoport, 2005; Gunawardena, 2014). These tools describe interactions between many biological components and emergent dynamics due to the complex relationships between them. Can these tools be integrated into the modeling of complex cell-free systems?

**Figure 1:**
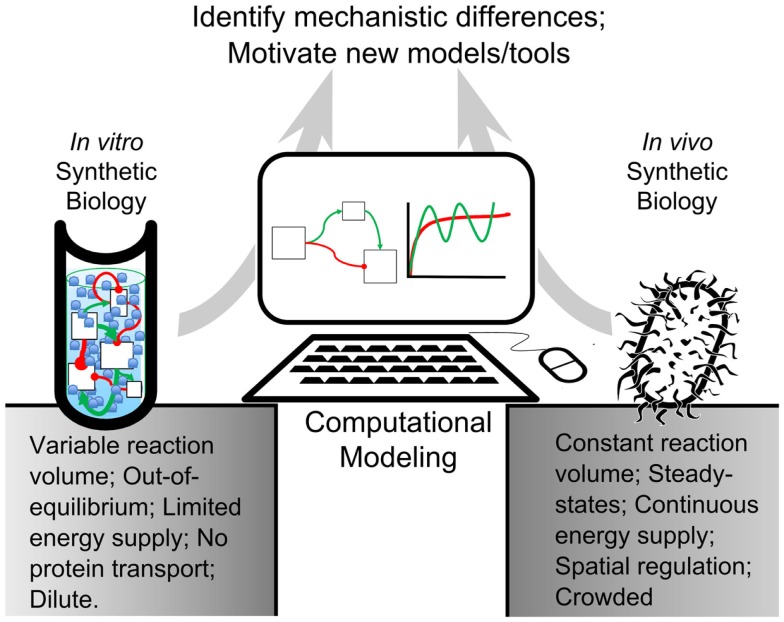
**Modeling-based inquiry of cell-free and *in vivo* synthetic systems**. An abstracted synthetic pathway (boxes and arrows) is modeled on a computer. The simulated expression dynamics are compared to biological and cell-free iterations of the process of interest. By using computational models to establish quantitative differences between *in vitro* reactions and *in vivo* systems, we could identify mechanisms in living organisms that contribute to desirable network behavior. These mechanisms could be added to *in vitro* reactions, bestowing useful properties on their processes. This way, computational modeling would bridge the gap between *in vitro* and *in vivo* reactions.

In this review, we present computational tools created for both *in vivo* and *in vitro* systems that are validated experimentally. In accordance with synthetic biologists’ goal of composing biological components into rational arrangements, we seek to bridge the gap between our understanding of complex biological networks and fundamental biochemical processes by comparing modeling algorithms for both systems (Figure 1). We will also examine some of the challenges that researchers face when engineering artificial cells. We intend to propose a framework for synthetic biologists to build novel artificial cellular systems and to identify underserved research areas for computational model development.

## Modeling Algorithms

### Deterministic modeling

Deterministic models typically consist of differential equations that predict the kinetics of a biological network based on past dynamics of the system and its initial conditions (Di Ventura et al., 2006). Deterministic models have been used to simulate synthetic gene networks, including inverters (Yokobayashi et al., 2002; Karig and Weiss, 2005), switches (Michalowski et al., 2004; Collins et al., 2006; Ham et al., 2008), band-pass filters (Basu et al., 2004; Sohka et al., 2009), multi-cellular networks (You et al., 2004; Basu et al., 2005; Tabor et al., 2009; Danino et al., 2010), and oscillators (Elowitz and Leibler, 2000; Stricker et al., 2008). Deterministic models have also been applied to simulate the behavior of tumor-invading bacteria (Danino et al., 2012), prokaryotic circuits capable of producing artificial analog computation (Daniel et al., 2013), and a transcriptional oscillator that retains its period across a range of temperatures (Hussain et al., 2014). These models utilize Michaelis–Menten equations to describe each chemical reaction.

For the modeling of *in vivo* systems, a baseline level of expression is typically included to model leaky activity of promoters. The leaky expression is attributed to the relationship between transcription factor binding strength and the corresponding RNA polymerase-promoter affinity. The typical model also assumes that all parameters are constant, which may not be true *in vivo* due to fluctuating states of intracellular environments.

In contrast to *in vivo* systems, cell-free systems could offer greater predictability by having well-defined parameters, easily controlled inputs, and fewer unknown interactions. Therefore, cell-free systems may be more accurately simulated than *in vivo* reactions using deterministic models. These cell-free systems can perform many of the same functions of natural organisms with circuits including oscillators (Kim and Winfree, 2011; Montagne et al., 2011; Weitz et al., 2014), switches (Kim et al., 2006; Padirac et al., 2012), and logic elements (Takinoue et al., 2008). A recent work modeled *in vitro* expression by accounting for the rate of green fluorescent proteins (GFP) maturation, which is often ignored for *in vivo* models (Stogbauer et al., 2012). Constructing computational models of *in vitro* systems can also provide insights into the effects of network architecture on the dynamic behavior of genetic circuits. Previous work has shown that biological pathways can achieve oscillatory behavior via bi-stable, hysteretic loops, and demonstrated *in vitro* that these mechanisms could be used in living systems to control the transition to the mitotic phase in *Xenopus* embryogenesis (Hasty et al., 2001; Pomerening et al., 2003). Subsequent research into the modeling of synthetic *in vitro* transcriptional oscillators was used to determine the optimum system parameters required for sustained circuit behavior and to identify the network’s period and amplitude limitations (Kim and Winfree, 2011). Recently, this same model was applied to simulate the behavior of an *in vitro* oscillator after compartmentalization in emulsion droplets and was found to accurately represent the trend observed in individual encapsulated circuits (Weitz et al., 2014).

**Figure 2:**
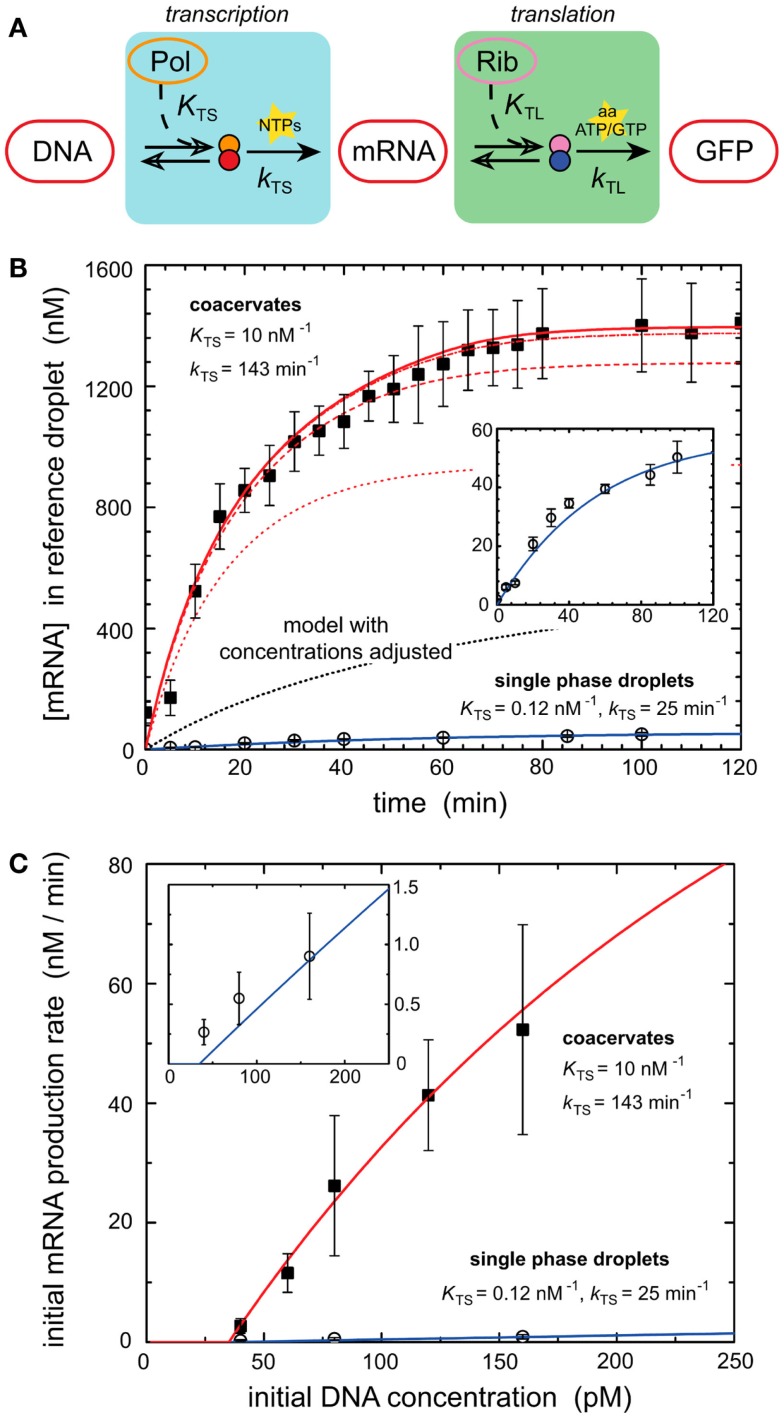
**Enhancement of transcription by coacervation**. **(A)** A schematic of biochemical processes that produce GFP from a DNA template via transcription and translation. Key reactants such as NTPs, amino acids, ATP, and GTP are depicted at their active steps. **(B)** Graph of mRNA production (nanomoles) over time from a genetic module encapsulated in coacervates and droplets. Dark squares represent experimental results for coacervate reactions. Open circles represent experimental results for droplet reactions. Lines represent deterministic simulations of the genetic module. Solid blue line corresponds to simulation of droplet reaction. Inset is a re-scaled view of the same experimental and simulated results of droplet reaction. The dotted black line represents a deterministic model of GFP production in coacervates with adjusted concentrations of transcription machinery. Red lines represent simulation results using altered T7 RNA polymerase binding constant *K*_TS_ and transcription rate constant *k*_TS_. The series of red lines represents simulation results for a constant *k*_TS_ of 143 min^−1^ and different values of *K*_TS_, 0.12 nM^−1^ (dotted), 1.0 nM^−1^ (dashed), 10 nM^−1^ (dash-dot), and 100 nM^−1^ (solid). Predicted values of *K*_TS_ and *k*_TS_ are listed for both coacervates and single phase droplets. **(C)** Initial rates of mRNA production as dictated by the initial concentrations of DNA (picomoles), which contain the genetic module. Dark squares represent experimental results for coacervate reactions. Open circles represent experimental results for droplet reactions. The red line represents simulation of coacervate with altered *K*_TS_ and *k*_TS_ parameters established in **(B)**. The solid blue line represents simulation of droplet reaction. Inset is a re-scaled view of the same experimental and simulated results of droplet reaction. Figure modified with permission fromSokolova et al. (2013).

Models of *in vitro* systems are also used to explore the impact of biological phenomena that are missing in reconstituted systems. Molecular crowding represents such a phenomenon and has been incorporated into models of gene expression to explain some of the desirable properties of biological systems. Recent work demonstrated that molecular crowding, either induced by crowding agents such as dextran or coacervation of encapsulated circuits can greatly increase the expression rate and total protein production of *in vitro* systems (Sokolova et al., 2013; Tan et al., 2013). One of these works investigates the mechanisms of enhanced transcriptional output induced by coacervation of encapsulated cell lysate (Sokolova et al., 2013). Coacervation was induced by treating encapsulated cell lysate with a concentrated salt solution, drawing water out from the droplets by osmotic pressure (Sokolova et al., 2013). Protein synthesis was dramatically increased within coacervated systems (Sokolova et al., 2013). A computational model of transcription–translation reactions revealed that the rise in mRNA output could not be explained simply by the increase in density of transcription machinery induced by coacervation (Figure 2) (Sokolova et al., 2013). The model instead suggested that the increase in gene expression was due to an elevated kinetic transcription constant in coacervated compartments, and a rise in the T7 polymerase association constant caused by increased molecular crowding (Sokolova et al., 2013).

### Stochastic modeling

To investigate the impact of random fluctuations, stochastic models of cellular processes can be formulated following the master equations. For *in vivo* systems, noise arises due to intrinsic and extrinsic factors (Elowitz et al., 2002). Extrinsic noise is variation caused by incomplete distribution of reactants within a system, whereas intrinsic noise is variation caused by the discrete nature of small-scale chemical reactions (Elowitz et al., 2002). Both kinds of noise can have a profound impact on biological systems, including partitioning noise observed during replication (Huh and Paulsson, 2011a), variance observed at small reaction volumes within a cell (Karig et al., 2013), and bursts of translation caused by limited transcriptional activity (Pedraza and Paulsson, 2008). Stochastic models have been applied to understand sporulation dynamics of *Bacillus subtilis* (Chastanet et al., 2010), robustness of a genetic circuit in response to varying environmental conditions (Toni and Tidor, 2013), exoprotease levels in bacterial populations (Davidson et al., 2012), and control of a bacterial population composition with a gene circuit (Sekine et al., 2011; Ishimatsu et al., 2013).

*In vitro* systems are minimal, which should intuitively simplify the development of computational models based on our experience in cell biology. However, due to this minimality, *in vitro* systems do not inherently contain mechanisms of natural cells that facilitate robust behavior. These missing mechanisms could increase sensitivity of *in vitro* systems to non-genetic factors, such as partial degradation products (Kim and Winfree, 2011), stochastic variation at femtoliter volumes (Karig et al., 2013), and molecular crowding (Tan et al., 2013). These factors, while commonly being neglected in models of natural cells, could reduce predictive power of models for cell-free systems. In addition, *in vitro* systems lack cellular infrastructure, including sub-cellular compartments, transport proteins, and a replication cycle. These missing cellular features could complicate the direct application of computational tools created for natural cells to *in vitro* systems, necessitating the development of stochastic models to predict and control noise in cell-free systems.

**Figure 3:**
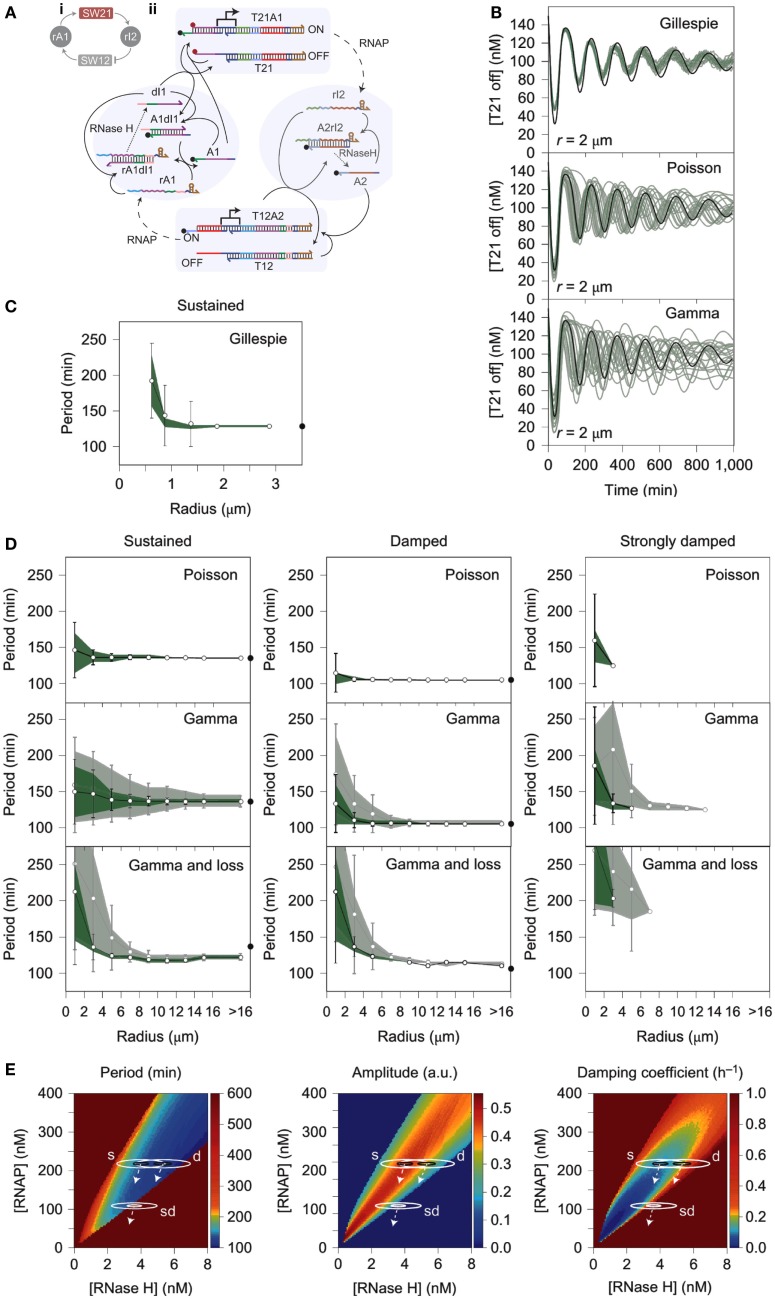
**Stochastic simulation of a compartmentalized oscillator**. **(A)** A linear construct (T21A1) transcribes a short RNA oligo (rI2) that base-pairs with a nicked region of another linear DNA promoter (T12A2). Base pairing of rI2 with the nicked DNA region A2 deactivates T12A2 because T7 promoter region of the switch becomes single stranded, referred to as T12. Deactivation of the T12 construct reduces transcription of RNA oligo rA1, which base pairs with nicked DNA region A1 of the T21A1 construct. Base pairing of rA1 with dl1 causes A1, a quencher labeled-strand of DNA, to separate from T21. T21 also has a fluorescent labeled strand of DNA. This reaction activates the fluorescent signal, but deactivates transcription of RNAP because the T7 promoter of T21 becomes single stranded. Essentially, the oscillator is formed by the T21A1 module (SW21) that inhibits the T12A2 module (SW12), which in turn activates SW21. The delayed negative feedback loop causes a temporal delay between ON and OFF cycles of T21A1. **(B)** The panels show concentrations of T21 in the OFF position (low fluorescence, high transcriptional activity) over time according to the Gillespie algorithm, Poisson Distribution, and Gamma Distribution, respectively. Droplet radius is always 2 μm. **(C)** The oscillator period is plotted against the radius of the compartment housing the *in vitro* circuit. The results suggest that only a small percentage of the variability experienced by the system can be attributed to the intrinsic noise of biochemical reactions. **(D)** Fluctuations of the oscillator period with increasing radius of the compartment. Panels show partitioning variance (error caused by incomplete distribution of reactants) of period following a Poisson distribution, Gamma distribution, or a Gamma distribution that accounts for loss of enzyme activity. Dark green shading corresponds to a scale factor β = 10, and light green shading corresponds to β = 100. **(E)** Phase diagrams that indicate period, amplitude, and dampening coefficient as a function of RNAP and RNase H concentrations. Solution space that achieves sustained oscillations, dampened oscillations, and severely dampened oscillations are referred to in the diagram as s, d, and sd, respectively. The white arrows represent behavioral trends experienced by the circuit when it loses RNAP and/or RNAse H. Figure modified with permission from Weitz et al. (2014).

One notable cause of stochastic variation in cell-free expression is the process of encapsulation, which could be simulated using stochastic models. For instance, during the compartmentalization of the PURE system in small liposomes (580 nm > diameter > 35 nm), the distribution of reactants between compartments was shown to follow power law statistics instead of a Poisson distribution as previously assumed (Lazzerini-Ospri et al., 2012). A subsequent study of *in vitro* systems encapsulated in larger liposomes (575 nm and 2.67 μm, respectively) predicted resulting reactant concentrations via a stochastic model following the Gillespie algorithm (Calviello et al., 2013). Another example of stochastic variation in an encapsulated *in vitro* system comes from a recent work detailing the behavior of a compartmentalized transcriptional oscillator (Weitz et al., 2014). The performance of the circuit within an emulsion was highly variable and was originally assumed to be the result of intrinsic noise of the system acting stochastically at small volumes (Weitz et al., 2014). However, the model of the reaction demonstrated that intrinsic noise was insufficient to describe the variability exhibited by the system; instead, the dominant cause of the deviation from the deterministic model was more likely to be extrinsic noise caused by heterogeneous distribution of reactants within the emulsion (Figure 3) (Weitz et al., 2014). This kind of discrepancy from the deterministic model is also observed during replication when cytoplasmic components are unequally distributed between daughter cells (Huh and Paulsson, 2011a,b; Weitz et al., 2014). The significant impact of extrinsic noise on this minimal system suggests that reactant distribution is an important factor in encapsulated *in vitro* reactions, which could be overlooked when considering the source of stochastic variation within *in vivo* systems (Huh and Paulsson, 2011a,b; Weitz et al., 2014).

The study of molecular crowding revealed how molecular distribution can impact stochastic variation *in vitro*. Molecular crowding has long been recognized as an important phenomenon for accurately reconstituting the function of biological systems, and recent research efforts have demonstrated that molecular crowding increases expression levels *in vitro* (Minton and Wilf, 1981; Morelli et al., 2011; Sokolova et al., 2013; Tan et al., 2013). Stochastic models of *in vitro* systems have also revealed decreased variation of gene expression rates in the presence of molecular crowding conditions, along with increased robustness to environmental perturbations of molecules known to affect the binding affinity of transcription–translation machinery (Tan et al., 2013).

### Exploratory models

Exploratory modeling is used to guide the design of biological circuits. To emulate labor-saving strategies from the engineering disciplines, there has been a push for automated biological design, combining known modules into more complicated architecture (Cheng and Lu, 2012). An automated design algorithm first registers a library of biochemical parts with defined kinetic parameters and interactions described in terms of ordinary differential equations (Marchisio and Stelling, 2008). Next, the algorithm selects certain parts and arranges them into motifs that satisfy a user’s query (Marchisio and Stelling, 2011; Beal et al., 2012; Huynh et al., 2012; Yaman et al., 2012; Huynh et al., 2013). Exploratory models can also be used to analyze the impact of intrinsic noise, extrinsic noise, and variation of kinetic parameters on synthetic genetic machinery (Chiang and Hwang, 2013; Toni and Tidor, 2013). These exploratory models have been used to design *in vivo* pathways, such as a Boolean network of transcriptional switches implemented in yeast (Marchisio, 2014), a multiplexor circuit in *E. coli* (Huynh et al., 2013), and an inducible bi-stable system of fluorescent reporters in mammalian cells (Beal et al., 2012).

In theory, these automated genetic design programs could be applied in the development of *in vitro* expression systems. On the one hand, transcriptional networks *in vivo* and *in vitro* have the same circuit architecture and basic components. Furthermore, the parameters used by these automatic genetic design programs are actually determined *in vitro*, which would make the assembly of *in vitro* circuits more accurate than *in vivo* circuits. On the other hand, genetic design programs optimized for *in vivo* conditions may not account for the chemical conditions experienced by *in vitro* expression systems. For instance, cell-free expression systems have limited substrates, contain molecular components with different reaction efficiencies than their *in vivo* counterparts, and do not inherently contain complex mechanisms (such as metabolic feedback loops, assisted protein folding, and protein trafficking) that maintain robustness of cellular functions.

### Parameter definition

While there are a wide variety of equations that describe the behavior of synthetic biological systems, parameters of these equations are mostly unknown. There are several established databases for obtaining enzymatic reaction constants such as KEGG (Kanehisa and Goto, 2000; Kanehisa et al., 2014), BRENDA (Schomburg et al., 2013), SABIO-RK (Wittig et al., 2012), and ExPASy (Artimo et al., 2012). BioNumbers has also collected measurements of biological systems (Milo et al., 2010) and has been used in the modeling of a yeast–bacteria ecosystem (Biliouris et al., 2012), a predictor of anti-microbial protein efficacy (Melo et al., 2011), and a computational representation of distributive metabolic networks (De la Fuente et al., 2013). The difficulty of modeling *in vivo* systems stems from the context-dependency of reaction parameters. The kinetic constants of biological molecules used in modeling *in vivo* systems are often measured *in vitro*, where conditions may not reflect the pH or molecular crowding conditions experienced by those molecules in natural cells. In contrast, these kinetic constants that are quantified *in vitro* could be directly applied to cell-free reactions, thus creating models with high accuracy and predictive power. We compare kinetic constants determined for the two dominant *in vitro* expression platforms: whole cell extracts and the PURE system (Table 1). The table contrasts the transcription rate constant, translation rate constant, and mRNA degradation rate. The transcription rate is slightly higher in the PURE system, but the whole cell extract has a significantly higher translation rate and mRNA degradation rate. These results suggest the presence of critical factors in whole cell extracts that have not been fully reconstituted in the PURE system. We note that the two studies used different genes, suggesting potential follow-up work to systematically compare and model different *in vitro* systems.

**Table 1:** **Expression constants for *in vitro* systems**.

	Whole cell	PURE expression
	extract	system
*k_p_* transcription (rNTPs/second)	1.0 ± 0.05^a^	2.2^b^
*k_r_* translation (amino acids/second)	>4^a^	0.03^b^
*k_d_* mRNA degradation rate (per second)	1.38e−4^b^	1.31e−5^b^

*^a^Karzbrun et al. (2011)*.

*^b^Stogbauer et al. (2012)*.

Now that we have outlined the broad classes of models available for synthetic biologists and identified some areas of potential growth for researchers interested in developing tools for cell-free systems, we will discuss the specific applications of computational tools to the design of an *in vitro* gene expression platform known as the artificial cell.

## The Components of Artificial Cells

**Figure 4:**
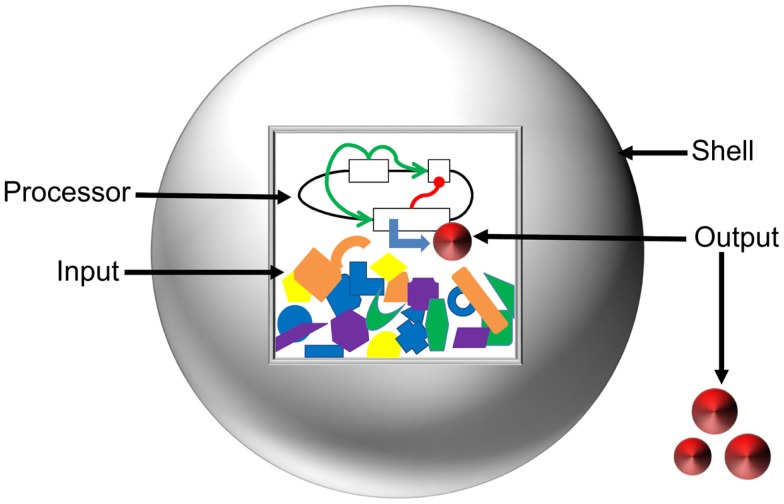
**Anatomy of artificial cells**. Input and Processor are contained inside the liposome Shell (gray sphere). The Inputs, represented by orange, blue, purple, yellow, and green shapes, are the energy supply, enzymatic co-factors, and substrates necessary for gene expression. The Processor is the cellular circuit (represented by black lines and white boxes) that controls protein production. The Processor determines the network architecture of cellular components (functional relationships represented by green and red lines) and the machinery required to interpret the information. The action of the Processor on the Input produces the Output (red disks) in the form of either a metabolite or a protein. The Shell serves to modulate diffusion of signals from the environment, maintain chemical conditions favorable for gene expression, and allow for export of the Output.

To layout the vision toward a comprehensive model of artificial cells, we have classified the system into the Input, Processor, Output, and Shell (Figure 4). Here, we define the Input as the starting concentrations of enzymes, metabolites, and inducers that are present in a system. The Processor is defined as the cellular circuit that dictates genetic composition and functional relationship between genes. The Output is described as the concentration of the final product(s) of a system. The Shell refers to the liposome barrier that controls interaction between artificial cells and the environment. We discuss mathematical design and optimization of each component and assemble a suite of computational tools that could be applied for predictive modeling of artificial cells.

## Input

The Input is defined as the co-factors, substrates, and chemical energy used in the execution of *in vitro* reactions. Although these factors are known to significantly alter gene expression, concentrations of these molecules are difficult to be perturbed *in vivo*, making their effects on gene expression elusive (Jewett et al., 2008, 2009). Mathematical modeling offers a unique solution to this problem by providing a formalized framework to evaluate the accuracy of a predicted Input concentration. A few publications have sought to quantify the impact of the Input on gene expression in cell-free systems. However, given the intricate complexity required to produce proteins *in vitro*, there is still substantial room for innovation. One computational model was created to determine the epistatic interactions between 69 elements of an *in vitro* transcription–translation system (Matsuura et al., 2009). Another model utilized a machine learning algorithm to stochastically vary different components of the Input (Caschera et al., 2011). Reaction systems with the highest expression levels were found to have two primary expression patterns: one with a rapid rise of protein production and the other with an initial decrease in protein level before a rapid rise (Caschera et al., 2011). A stochastic model of an *in vitro* transcription–translation system was used to vary reaction components such as potassium, magnesium, and spermidine to investigate the impact of environmental perturbations on gene expression (Tan et al., 2013). To aid the development of subsequent models that describe the effects of the Input on gene expression *in vitro*, a recent work has compared physiological and supplemented concentrations of intracellular components within cell-free systems (Jewett et al., 2008).

## Processor/Output

The Processor is defined as the DNA sequence that dictates genetic composition and functional relationship between genes, together with the machinery required to interpret it [RNA polymerases (RNAPs), transcription factors (TFs), translation machinery]. The Output is defined as the final product of a system. In the context of this review, we define Output as the product of the activity of the Processor (mRNA for transcription systems, protein for coupled transcription–translation systems, and metabolites for enzymatic reactions). We will review Processor and Output together because they are required for the integral understanding of gene expression. These modules are critical to connect input signals to functions of synthetic biological systems. In the following sections, we will review some of the strategies developed to predict activities of Processor modules and to control the expression of genes. Many of these approaches have been validated primarily in natural cells, but could be adapted for cell-free systems.

### Sequence-based control of promoter transcriptional activity

**Figure 5:**
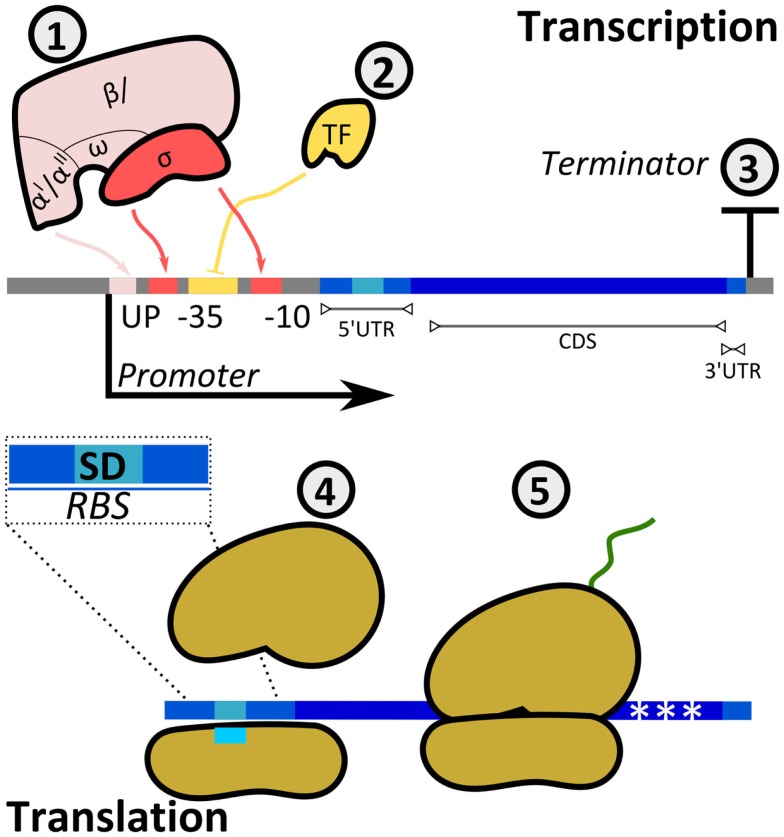
**Regulatory mechanisms of protein production rates**. The synthesis rate of a protein can be controlled at either transcriptional (1, 2, 3) or translational (4, 5) levels. (1) The multi-subunit bacterial RNAP interacts with the promoter at boxes −35 and −10 (σ-factor) and at the UP element (α-subunits). The sequence of the promoter defines the specificity and affinity of the binding. (2) Transcription factors (TFs) either enhance or inhibit the binding of RNAP to the promoter (in the example, TF is a repressor inhibiting RNAP–DNA interactions). TF–RNAP–DNA interactions determine the initiation of transcription. The synthesized mRNA contains a 5′-untranslated region (UTR), a coding sequence (CDS), and a 3′-UTR. (3) The terminator sequence determines RNAP release from DNA. (4) Translation initiation is the rate limiting step for translation. It is determined by interactions between the ribosomal binding site and the small ribosomal subunit, with specific heteroduplex mRNA:rRNA formation between the Shine–Dalgarno (SD) sequence in the transcript and the 16S rRNA. In addition, codon usage (5) can affect the amount of translated protein.

Cells have evolved mechanisms to regulate both transcription and translation rates to adjust the expression levels of target proteins (Figure 5). The promoter region allows the binding of RNAPs to DNA to initiate the synthesis of mRNA. Prokaryotic RNAPs typically bind promoters through the specific recognition of their σ-subunits to conserved sequences at the −10 and −35 promoter positions (transcription starts at position + 1), while RNAP α-subunits interact with elements located upstream of the −35 site. Furthermore, the activity of RNAP is modulated by activators and repressors. Following RNAP–DNA interactions, mRNA is synthesized until the RNAP reaches a terminator.

Several studies have established libraries of artificial promoters with different sequences, which are compared by measuring the accumulation of reporter proteins (Hammer et al., 2006; De Mey et al., 2007; Ellis et al., 2009; Rhodius and Mutalik, 2010; Lu et al., 2012; Rhodius et al., 2012; Temme et al., 2012; Iyer et al., 2013; Shis and Bennett, 2013). In some of these studies, the experimental data collected have been used to build inference models (Gunawardena, 2014), in order to establish causal relationships between promoter sequence and its strength. Next, we will describe approaches used to predict promoter strengths, which we define as the association rate constants of RNAP–promoter complex. Therefore, if the binding affinity of RNAP to a promoter is high, the promoter is “strong,” which increases Output accumulation. Conversely, a promoter is “weak” if the affinity between RNAP and promoter is low, resulting in a reduced transcription rate.

### Promoter strength can be predicted based on RNAP and transcription factors binding dynamics

Cellular biochemical functions, including gene transcription, have been grouped into defined subsets according to their approximated fitting to differential equations in the form of Hill functions. This approach is useful for predicting the activity of a promoter, which depends on the binding affinity of RNAP and regulatory TFs to DNA, the position of the promoter, and the position of other regulatory sequences in the promoter (Ang et al., 2013). For example, the transcription of a gene controlled by a promoter with binding sites for an RNAP and a TF can be expressed as Eq. 1 for an activator and Eq. 2 for a repressor.

(1) $$\frac{\mathrm{dy}}{\mathrm{dt}}=k^{\prime }+k\left(\frac{x^{n}}{K^{n}+x^{n}}\right)-k_{d}y$$

(2) $$\frac{\mathrm{dy}}{\mathrm{dt}}=k^{\prime }+k\left(\frac{K^{n}}{K^{n}+x^{n}}\right)-k_{d}y$$

For both Eqs (1) and (2), *y* is the Output (mRNA), *k_d_* represents the degradation rate constant. For Eq. 1, *k*′ is the basal rate of Output production associated to RNAP affinity for the promoter and *k* is the maximum production rate (measured by the energy binding of activator TF to RNAP). The bracketed term corresponds to a Hill function, where *x* is the concentration of the TF and *K* is the Hill’s constant corresponding to the binding affinity of TF to DNA. Finally, *n* is the Hill coefficient that indicates TF cooperative effect. For Eq. 2, *k*′ + *k* accounts for the expression rate in un-repressed conditions and corresponds to the binding affinity of RNAP to the promoter. In this case, the Hill function has a decreasing sigmoidal shape. In the absence of TFs (either activators or repressors), as for the case of constitutive promoters, the Output production will depend exclusively on the binding affinity of RNAP to DNA.

**Figure 6:**
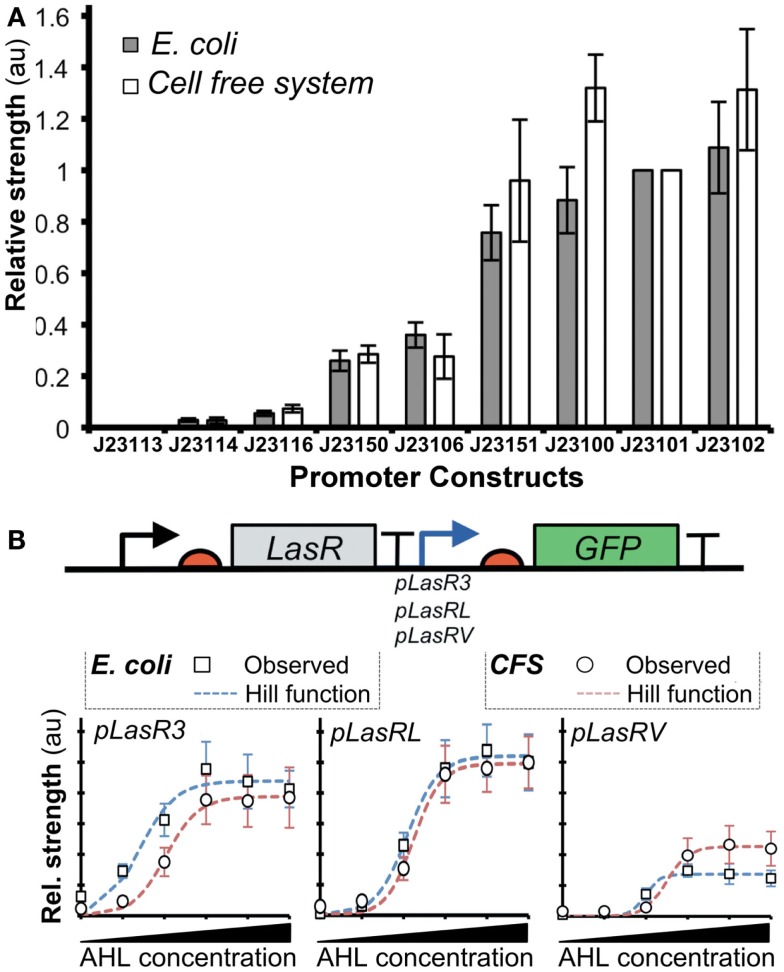
**Comparison of promoter strengths *in vivo* and *in vitro***. **(A)** Several promoters controlling expression of GFP showed comparable activities when assayed in cell-free systems and *E. coli*. The *x*-axis shows different promoter-GFP constructs. The *y*-axis shows relative promoter strengths measured by GFP intensities (au). **(B)** GFP expression is regulated by pLas promoters that are bound by an activator LasR with different affinities. Fluorescence intensities were determined for the constructs both *in vitro* (open circles) and *in vivo* (gray squares). A model of gene expression was built using the Hill function (red dashed lines represent cell-free systems; blue dashed lines represent *E. coli*). The result shows that Hill functions can be used for quantifying gene expression in cell-free systems. Figures modified with permission fromChappell et al. (2013).

A recent work described a novel method based on the Hill functions to characterize regulatory elements in cell-free systems (Chappell et al., 2013). In this study, the relative activities of σ^70^-promoters were demonstrated to correlate well between cell-free systems and bacterial systems (Figure 6A). Furthermore, they tested the activity of a promoter in the presence of an activator transcription factor, LasR (Figure 6B). After the addition of acylhomoserine lactone (AHL), LasR affinity for the promoter increases, allowing recruitment of RNAP and subsequent gene transcription. Output levels (GFP) of different LasR-regulated promoters were found to correlate well between *in vivo* and *in vitro* systems (Figure 6B). However, the authors observed that estimated RNAP binding efficiency did not correlate well between *in vivo* and *in vitro* systems. Several factors could account for the observed differences: (1) concentrations of macromolecules (i.e., proteins, DNA, and ribosomes), (2) ratios between proteins and other co-factors (proteins or enzymes), (3) structure and supercoiling of DNA, (4) molecular crowding (confinement vs. non-confined environment), (5) composition of energy and/or redox power regeneration systems. Due to these differences, it remains unclear when it is appropriate to extrapolate kinetic information between *in vitro* and *in vivo* systems. To this end, mathematical modeling could help to identify and potentially resolve the differences.

### Prediction of promoter strengths based on its nucleotide sequence

**Figure 7:**
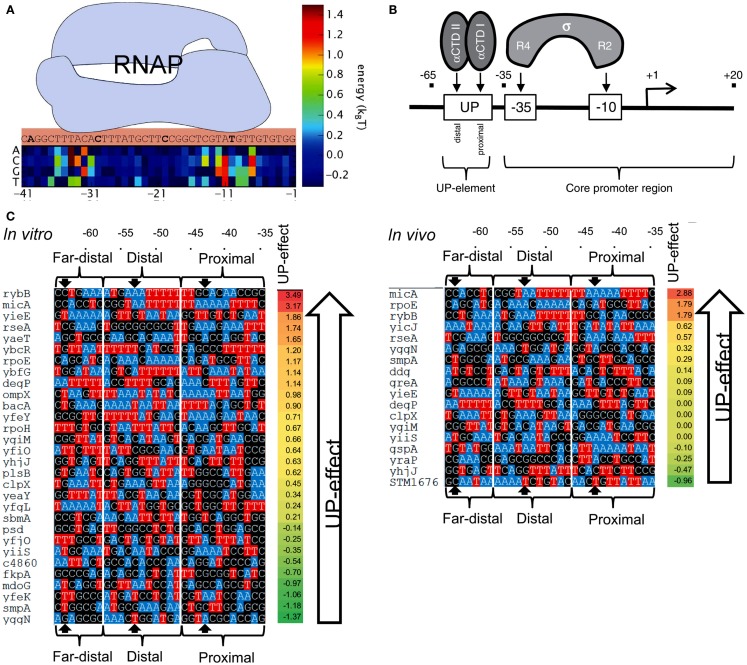
**Prediction of promoter strengths based on DNA sequences**. **(A)** The binding energy of RNAP (light blue shape) to a promoter can be estimated based on its DNA sequence. A position matrix defines the contribution of each base to the total binding energy. Bases at sites −10 and −35 are major contributors to the overall binding energy. At some positions, the occurrence of certain bases decreases RNAP binding energy. **(B)** The approach is used to determine the effect of UP elements on gene transcription. UP elements are recognized by the α-subunit of RNAP and located upstream of the −35/−10 sites (core). **(C)** The activities of several *E. coli* promoters containing an UP element (with three distinguished substrings, termed proximal, distal, and far-distal) were determined both *in vitro* (left) and *in vivo* (right), and compared to the activity of the core promoter (promoter lacking the UP element). The impact of UP elements on gene transcription was calculated by the ratio between the activity of a complete promoter (a core promoter plus an UP element) and the activity of the corresponding core promoter. The bases A and T are colored to indicate the AT tracts in the promoters. AT tracts are known to increase the influence of UP elements on gene transcription. Figures modified with permission from Brewster et al. (2012) and Rhodius et al. (2012).

In order to predict the strength of a given promoter sequence, extensive effort has been made to build models that describe the causal relationships between promoter sequences and their affinity to RNAP. In general, the binding energy of RNAPs to DNA has been considered to be a linear addition of the individual energy barriers of each base in the promoter sequence (thermodynamic-based models; Figure 7A) (Mulligan et al., 1984; von Hippel and Berg, 1986; Berg, 1988; Takeda et al., 1989; Stormo, 2000; Benos et al., 2002; Segal et al., 2008; Rhodius and Mutalik, 2010). However, inconsistencies have been observed between models and experimental observations. For example, several promoters were predicted to exhibit strong transcription rates, but were either weak or inactive *in vivo* (false positives) (Stormo, 2000; Man and Stormo, 2001; Rhodius and Mutalik, 2010). A fraction of strong promoters were also not identified by the models (false negatives) (Maerkl and Quake, 2007).

Despite these drawbacks revealed by genome scale studies of promoter activity, additional models have been developed and used to predict bacterial RNAP-promoter activity *in vivo* based on their sequences. For example, a set of promoters with variable RNAP-σ^70^ binding sites were tested for their strengths, in terms of both transcriptional activity and activity of the encoded gene (β-galactosidase) (Brewster et al., 2012). The sequences of the promoters were chosen based on a predictive model, built with sequence-based position weight matrices and binding energies. The model predictions correlated strongly with observed promoter strengths *in vivo*. The model was also applied to promoters that were regulated by a repressive transcription factor, confirming that the model can be used for prediction of promoter strengths. In another study, a library of *Escherichia coli* lac promoters was generated (Kinney et al., 2010). The promoters contained mutations in binding sites of both RNAP-σ^70^ and CRP, as well as an activator TF. Promoters with both or either binding sites were assembled and their strengths were measured according to reporter GFP fluorescence intensity. RNAP–DNA, CRP–DNA, and RNAP–CRP interaction strengths determine the overall promoter strengths. The promoter strengths were estimated using a model based on position matrices for each binding site, which assigned a quantitative value to the influence of each position along the promoter on its activity. Through this strategy, the authors were able to model RNAP– and CRP–DNA binding strengths, and also RNAP–CRP interaction energy, to establish relationships between promoter sequences and transcription rates.

Predictive models of promoter strengths *in vitro* are less well established when compared to *in vivo* models. Rhodius et al. (2012) assessed strengths of promoters with mutations in −35/−10 sequences and the upstream UP elements (Figure 7B). Using a library of core promoters (containing regions −35/+20) and their corresponding full-length versions (core promoter plus UP element, −65/+20), the authors determined the activity of the promoters both *in vivo* (Output: GFP) and *in vitro* (Output: mRNA). They built a model to estimate the effects of the UP elements on promoter strengths. The authors built position weight matrices (PWMs) for each motif in the promoters (Stormo, 1990; Rhodius and Mutalik, 2010). The relative binding affinity of σ^E^ to a DNA sequence was estimated by adding the individual weights of each nucleotide in the motif. The scores of core promoters, UP elements, and full-length promoters were used to calculate the overall promoter strengths (Figure 7C). Based on the approach, the predicted and observed promoter strengths showed strong correlation between *in vivo* and *in vitro* systems.

### Theoretic basis for modeling T7 promoter activity for cell-free circuits

In cell-free systems and artificial cells, the use of bacterial RNAPs has been challenging due to their multimeric composition and low transcription efficiency. Instead, the use of monomeric, phage-derived RNAPs, such as T7-RNAP and SP6 RNAP, can simplify the design and application of *in vitro* genetic circuits. In addition, T7-RNAP is a highly processive polymerase, is not regulated by TFs, and binds to specific T7 promoters (Bintu et al., 2005). To date, no sequence-based and predictive models of T7 promoter strength have been published. To this end, predictive thermodynamic models for T7 promoter strengths could be developed following existing models for bacterial RNAPs.

Tremendous biophysical information is available for T7 RNAP and its associated T7 promoters. A collection of T7 promoters with different DNA sequence was tested *in vivo* (Imburgio et al., 2000) by quantifying mRNA as Output signals. In another study, T7 promoters with mutations in −11 to −8 bases were assayed using a split T7 RNAP, where C-terminal and N-terminal fragments were individually expressed (Shis and Bennett, 2013). When both fragments are expressed, T7 RNAP becomes functional (Output: GFP). Mutations were also introduced in the C-terminal fragment’s specificity loop of T7 RNAP, which gave rise to combination of promoters and T7-RNAP variants with a broad range of transcriptional activity. Recently, a library of 21 T7 promoters was characterized in cell-free platforms using an *in vitro* transcription–translation system (Chizzolini et al., 2013). In addition, detailed kinetic data of T7-RNAP–promoter interactions are available (Bandwar et al., 2002), as well as protein:DNA structural data (Cheetham and Steitz, 2000). Taken together, these data suggest that a sequence-based predictive model of T7 promoter strength could be developed and subsequently validated for *in vitro* control of gene expression levels.

### Control of translation initiation rate by modification of ribosomal binding sequence

Translation initiation rates can be controlled to modulate Output accumulation *in vitro* (corresponding to protein accumulation). The translation process involves three main steps, including initiation, elongation, and termination (Simonetti et al., 2009). In bacteria, the 16S rRNA (from the small ribosomal subunit 30S) interacts with the Shine–Dalgarno (SD) sequence present in the 5′-untranslated region (UTR) of mRNAs (Kozak, 1999) (Figure 5). The initiation complex is completed with the binding of initiation factors and the large ribosomal subunit 50S. Additional sequences upstream and downstream the SD sequence determine the initial translation rate (Espah Borujeni et al., 2014). These sequences together with the SD sequence are termed ribosomal binding site (RBS).

**Figure 8:**
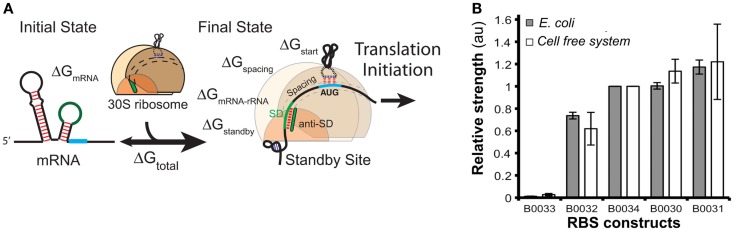
**Models of ribosome binding sites (RBS) and their potential applications for *in vitro* systems**. **(A)** A thermodynamic model for the calculation of RBS strength based on its sequence. The RBS calculator (Salis, 2011) is based on the calculation of the mRNA–ribosome binding energy (ΔG_total_). The sequence upstream of the Shine–Dalgarno (SD) site determines a penalty score ΔG_standby_ that is due to the work required to unfold secondary structures in this region. **(B)** The strengths of several RBS (controlling translation of GFP) were comparable when assayed both *in vivo* and *in vitro* [different RBS-GFP constructs in the *x*-axis, relative fluorescence (au) in the *y*-axis]. Figures modified with permission from Espah Borujeni et al. (2014) and Chappell et al. (2013).

Ribosomal binding site strengths can be predicted using multiple tools, including RBS calculator (Salis, 2011) (Figure 8A), UTR Designer (Seo et al., 2013), and RBSDesigner (Bujara et al., 2010). These tools compute differences of free energy between the folded secondary structures of a RBS (representing the state when mRNA is not bound to ribosomes) and its unfolded state (bound to the ribosome). The relative functionality and limitations of these RBS models were recently reviewed elsewhere (Reeve et al., 2014).

These tools have been successfully applied to fine-tune protein translation in natural cells. For example, RBS calculator was used to adjust translation rates of genes in the *nif* operon, which was responsible for nitrogen fixation in *Klebsiella oxytoca* (Temme et al., 2012). RBS calculator was also used to demonstrate that modification of RBS sequence altered protein levels, but not mRNA accumulation (Pothoulakis et al., 2013). UTR designer (Lim et al., 2013; Seo et al., 2014) and RBSDesigner (Lee et al., 2013) were used *in vivo* to adjust translation rates of genes in metabolic operons, build a predictive library of RBS strengths, and fine-tune accumulation of reporter genes in a light-inducible expression system. These results suggest that RBS-based models can be used to predict the accumulation of target proteins *in vivo*.

In contrast, there are few publications that evaluate RBS strengths in cell-free systems. A recent report (Chappell et al., 2013) showed that relative strengths of RBS are similar for *in vitro* and *in vivo* systems (Figure 8B). The authors measured GFP translation both in whole cells and in cell-free systems using several RBS sequences with different predicted strengths. Despite the differences in the *in vivo* and *in vitro* biochemical environments, the accumulation of Output (GFP) was comparable for each tested RBS. These results suggest that RBS strengths *in vitro* could be estimated using existing tools developed for *in vivo* systems likely because RBS models rely solely on RBS sequence and secondary structures, as well as interactions between RBS and ribosomes.

### Other factors influencing gene output accumulation

The control of transcription and translation initiation accounts for most of the common strategies used to control accumulation of target proteins. However, other factors could be considered to improve target accumulation at desired levels. Terminators are necessary to promote detachment of RNAP from DNA and release of the synthesized RNA (Figure 5). In the absence of efficient terminators, the RNAP will continue transcribing throughout the DNA, reducing the pool of RNAP available to initiate productive transcription rounds. Intrinsic terminators are recognized by RNAP without requirement of additional factors. Several biophysical models have been developed to estimate strengths of intrinsic terminators using solely their DNA sequence (Carafa et al., 1990; von Hippel and Yager, 1991; Cambray et al., 2013). Recently, a library of more than 500 terminators was characterized in *E. coli*, and the strengths of the terminators agreed with predicted strengths based on a simple thermodynamic model (Chen et al., 2013). Similar to bacterial RNAP, T7 RNAP recognizes a specific terminator sequence, which functions at low termination efficiency of 50–70% (MacDonald et al., 1993). Recently, T7-RNAP terminators with efficiencies of up to 99% have been developed (Mairhofer et al., 2014). Although these models were tested *in vivo*, the fact that intrinsic terminators do not require additional factors suggests that they could potentially be applied to cell-free systems.

Two other factors can also control Output accumulation. First, translation efficiency can be affected by the target gene sequence (Figure 5), affecting the concentrations of synthesized proteins. For example, codon usage can be specifically designed and optimized for a particular host or *in vitro* system (Chung and Lee, 2012), maximizing protein production. Second, the activity of a promoter and RBS depends on upstream and downstream sequences (Kammerer et al., 1986; Leirmo and Gourse, 1991; Salis et al., 2009; Espah Borujeni et al., 2014). Therefore, the implementation of insulator sequences that “buffer” the effects of surrounding sequences over the regulatory sequences (Davis et al., 2011; Mutalik et al., 2013) should be considered when designing cell-free systems.

### Metabolites as output: Prediction and control of metabolic pathways

Biocommodities are metabolites that have high economic values, including antibiotics, chiral compounds, and proteins (Zhang, 2010). Typically, biocommodities are produced using microorganisms with engineered metabolic pathways. However, the complexity of the biosynthetic pathway of interest can be reduced by isolating it from cellular metabolic network and engineered specifically to produce the desired target at determined rates. The decrease in the complexity of the cell-free isolated metabolic pathway could potentially lead to improved control over the system behavior and simplified purification of the target metabolite. *In vitro* production of metabolites could also avoid potential toxicity associated with synthesizing a biocommodity *in vivo*. Furthermore, theoretical calculations of product-to-biocatalyst weight ratios (total turnover number, TTN_W_) show that *in vitro* systems achieve TTN_W_ at several orders of magnitude higher than microbial-based production (Zhang, 2010), likely due to the removal of non-essential metabolic pathways. Indeed, theoretical calculations suggest that *in vitro* systems could be important tools for the production of biocommodities such as ethanol and butanol (Welch and Scopes, 1985; Zhang et al., 2010). Cell-free systems have been shown to efficiently produce metabolites (Bujara et al., 2010) and proteins (Calhoun and Swartz, 2005). For example, the granulocyte macrophage colony-stimulating factor, a multi-sulfide bonds protein, was produced at scales ranging from 250 μL to 100 L using synthetic expression systems (Zawada et al., 2011). In this context, the development of models that accurately predict productivity of *in vitro* systems could improve synthesis of biocommodities.

Several tools are available for the design of cell-free metabolic pathways. Public access databases such as KEGG (Kanehisa et al., 2014), MetaCyc (Caspi et al., 2014), ChEBI (de Matos et al., 2010), and RHEA (Alcantara et al., 2012), are useful for the design of metabolic pathways using parts from different organisms. BRENDA (Schomburg et al., 2013), a database containing molecular and biochemical data of enzymes, can be useful to select the core pathway that will produce the metabolite of interest. Web servers, such as From-Metabolite-To-Metabolite (FMM) (Chou et al., 2009) and Metabolic Route Search and Design (MRSD) (Xia et al., 2011), can also be used for designing synthetic and unique metabolic pathways in cell-free systems. Metabolic Tinker (McClymont and Soyer, 2013) can be used to identify and rank thermodynamically favorable pathways between two compounds, which may include novel, non-natural pathways. The XTMS platform (Carbonell et al., 2014) can help to rank pathways based on enzymatic efficiency and maximum pathway yields.

Together with the definition of metabolic pathways, it is important to establish the relative contribution of each enzyme to the accumulation of the target metabolite. Flux balance analysis (FBA) is commonly used to calculate the relative contribution of each enzymatic step in the pathway when optimization of particular objective function is required (Orth et al., 2010). FBA is based on the stoichiometry of the metabolic pathway and requires the selection of constraints to limit the solution space (Gianchandani et al., 2010). The system has to be solved for the maximization (or minimization) of an objective function, including metabolic fluxes, metabolite production, and biomass production. Several computational tools are available to solve FBA (and its variants, see Gianchandani et al., 2010), such as COBRA toolbox for MATLAB (Schellenberger et al., 2011) and the open-source version COBRApy (Ebrahim et al., 2013). Therefore, FBA can be useful for determining enzymatic steps and the required concentrations of the enzymes in the pathway. Thus, the combination of these tools will be valuable for designing cell-free systems as biocommodity production factories.

## Shell

The Shell is defined as the barrier that isolates the Input and the Processor from the environment. The diameter of the Shell can influence the degree of molecular crowding and reaction rates of the Processor. Furthermore, the Shell controls the import of signals from the environment and export of Output compounds from intracellular space of artificial cells.

**Figure 9:**
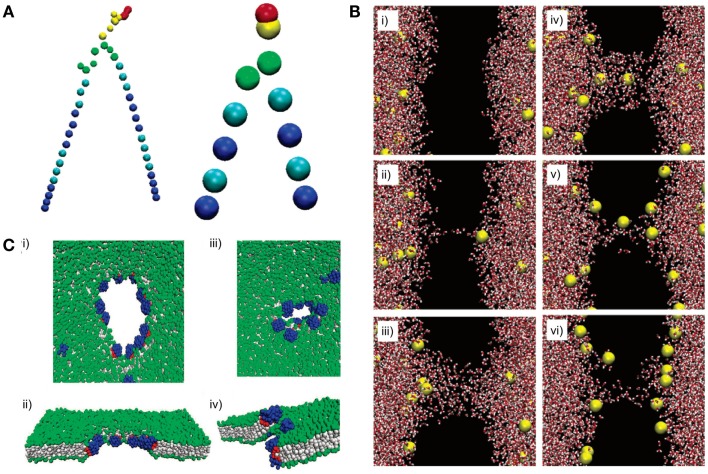
**All-atom (AA) and coarse-grained (CG) models can reveal lipid dynamics at different length and time scales**. **(A)** A DPPC lipid is represented with an AA model (left) and a CG model (right). All atoms are explicitly simulated in the AA model. In contrast, atoms are simplified into “beads” in the CG model. **(B)** A pore formation and closure event induced by ionic charge imbalance is simulated using an AA model. The dynamic is illustrated at different time points from (i) to (vi). Time points: (i) 20 ps, (ii) 450 ps, (iii) 1000 ps, (iv) 1070 ps, (v) 9180 ps, and (vi) 60 ns. The lipid bilayer is not shown (black space). Red and white shapes represent water molecules. Yellow shapes represent sodium ions. **(C)** Pore formation on a lipid membrane by nanoparticles is simulated using a CG model. (i–ii) The pore is formed and opened by applying an external stress. (iii–iv) Closure of the pore after the stress is removed. Gray shapes represent lipid tails. Green shapes represent lipid headgroups. Blue and red shapes represent hydrophilic and hydrophobic portions of the nanoparticle, respectively. Figures modified with permission from Gurtovenko and Vattulainen (2005), Baron et al. (2006), and Alexeev et al. (2008).

The advent of synthetic biology brings forth the desire to harness functioning principles of natural membranes for the control of the Shell of artificial cells. Natural membranes use many strategies, such as membrane proteins and lipid rafts to achieve information exchange with the environment (Klingenberg, 1981; Simons and Ikonen, 1997; Korade and Kenworthy, 2008). Currently, it is difficult to engineer artificial membranes to achieve the same complexity of natural membranes due to limited knowledge about the dynamics of lipid bilayers. To this end, computational tools have been implemented to close the gap between natural and artificial membranes by simulating dynamics of lipid bilayers and their interactions with the environment (Sum et al., 2003; Rog et al., 2007; Risselada and Marrink, 2008; Thakkar and Ayappa, 2010; Stepniewski et al., 2011). Here, we will discuss two types of molecular dynamic models, all-atom (AA) and coarse-grained (CG) models, which are categorized by the level of detail (Figure 9A). The degree of detail addressed by each algorithm is determined by force fields that make up these models. A force field consists of a set of mathematical functions and parameters that describe interactions between molecules. Development of various force fields is outside the scope of this review. Detailed reviews (Xiang and Anderson, 2006; Marrink et al., 2009) or comparisons (Baron et al., 2006; Siu et al., 2008; Perlmutter et al., 2011) of different force fields, such as CHARMM, GROMOS, AMBER, and MARTINI can be found elsewhere. This section of the review presents some examples on how computational modeling can boost our understanding of membrane behavior and dynamics, which could provide important insights into efficacious design of the Shell.

### All-atom models

All-atom models are useful tools in lipid membrane simulation. In AA models, every atom of the solute and solvent in the system is explicitly simulated. Thus, when applied to the simulation of lipid bilayers, AA models can provide fine details at the molecular level. Due to the computational cost, AA models are often limited to small-scale simulations (Xiang and Anderson, 2006; Marrink et al., 2009; Perlmutter et al., 2011).

All-atom models have been applied to simulate membrane defection by an electrical field (Tieleman, 2004; Bockmann et al., 2008) and pore-forming agents (Jean-Francois et al., 2008). For instance, Bennett et al. demonstrated spontaneous pore formation by restraining a single phosphate group at the center of the lipid bilayers. The simulated results agreed with previous computational (Tieleman, 2004; Bockmann et al., 2008) and experimental work (Paula et al., 1996). With tremendous detail at the atomic level, the simulation was able to reveal thermodynamics of transient pore formation and closure, which illustrated membrane defects from a novel perspective (Bennett et al., 2014). Similarly, Gurtovenko et al. used an AA model and illustrated that pore formation and closure could be induced by ionic charge imbalance (Figure 9B) (Gurtovenko and Vattulainen, 2005). Permeation of water or small solutes across lipid bilayers may be attributed to transient membrane defects (Deamer and Bramhall, 1986). The understanding of the dynamics of pore formation could be exploited to modulate the rates of molecular diffusion across membranes of artificial cells. This understanding could enhance our control of the activation of Processor by environmental signals and the rates of Output release from artificial cells.

### Coarse-grained models

In contrast to AA models, CG models are simpler and contain fewer details. Instead of explicitly describing every atom in the system, CG models consist of “beads,” which represent groups of atoms, potentially reducing the resolution of the simulation and decreasing computer resources required to simulate AA models (Baron et al., 2006; Marrink et al., 2007; Marrink et al., 2009). As a result, CG models are preferable when simulating large scale dynamics where atomic details may not be critical. For example, CG models have been used to simulate lipid phase behaviors, such as phase separation and phase transition (Risselada and Marrink, 2008; Prates Ramalho et al., 2011). Lipid bilayers have several phases that are generally characterized by relative mobility of lipid molecules. Phase changes can alter mechanical properties of membranes, such as fluidity and rigidity. Indeed, a simulation study using a mesoscopic model suggests that phase separation could impact liposome fusion dynamics (Smith et al., 2007). Thus, phase behaviors may need to be considered when designing the Shell to achieve certain mechanical properties. In one study, Risselada et al. used MARTINI force field to simulate lipid phase behaviors in a ternary lipid system. The simulation demonstrated that a mixture of saturated, unsaturated lipids, and cholesterol could spontaneously segregate into different domains. The liquid-ordered (L_o_) domains consisted mostly of saturated lipid and cholesterol, whereas the liquid disordered (L_d_) domains consisted mostly of unsaturated lipid (Risselada and Marrink, 2008). This simulation had been validated by experiments (Veatch et al., 2004). In addition, the simulation suggested that cholesterol was the key driving force for the phase separation.

Coarse-grained models have also been used to study interactions between lipid bilayers and other molecules. Ramalho et al. investigated the effect of nanoparticles on fluid-gel transformation of lipid bilayers. Nanoparticles were shown to induce local disorder of a lipid bilayer and delay the transformation of the lipid bilayer from fluid to gel states (Prates Ramalho et al., 2011). Other computational studies have shown that amphiphilic nanoparticles (Alexeev et al., 2008) (Figure 9C) and nanotubes (Dutt et al., 2011) interact with lipid membranes to form controllable pores and channels. These simulations could be used in conjunction with AA models of pore formation to provide guidelines when designing a permeable Shell.

### Model tradeoffs and prospect

The choice of AA or CG model depends on the context of scientific questions. When detailed atom–atom interactions are not a concern, CG models are suitable to compromise the computational cost. However, as computational hardware and software continue to improve, it is possible to use AA models to describe dynamics over a longer time scale (Sodt et al., 2014). Some studies have also combined AA and CG models to achieve long, yet fine time-scale simulation (Thogersen et al., 2008; Perlmutter et al., 2011). Briefly, CG models are first used to perform large time-scale simulation and then switched to AA models by mapping “beads” to single atoms.

Most simulation tools are focused on dynamics of the lipid membrane itself. To date, models integrating the Shell and the Processor/Output modules have not been established. The main hurdle for the integration lies in the difficulty of linking physical concepts used in membrane modeling and chemical dynamics utilized in transcription–translation modeling. Recent studies have shown that liposomes can affect gene expression (Bui et al., 2008; Umakoshi et al., 2009). The models discussed in this section only consider microscopic (atomistic) scale of membrane dynamics, but integrated simulation may be necessary for predicting dynamics of artificial cells. Beyond the atomistic scale, mesoscopic (about 0.1–10 μm) models where individual molecules are CG to single fluid volume are potential options for simulation of lipid bilayers (Ayton and Voth, 2002; Goujon et al., 2008). Other options are hybrid models where atomistic scale information is obtained and then “transformed” to lower resolution representations to achieve simulations at larger time- and/or length-scales (Ayton and Voth, 2002, 2009). This transformation can be challenging due to the lack of direct links between micro- and macro-scale dynamics. A recent work has developed a framework and attempted to incorporate physical (spatial location and diffusion) and chemical (biochemical reactions) methods to simulate cellular functions (Loew and Schaff, 2001). To this end, we envision that computational modeling of interactions between lipid membranes and transcription–translation machinery will provide unique insights into robustness of gene expression and enhance our capacity to control artificial cells.

## Conclusion

In this review, we have outlined differences between *in vitro* and *in vivo* synthetic biological systems. Current cell-free expression systems lack the spatial arrangement, protein transport, and folding, as well as various non-DNA binding factors that modulate gene expression in living organisms. These qualitative differences between *in vivo* and *in vitro* reactions could produce quantifiable differences in dynamical behavior between the two systems, which would require different modeling approaches. Molecular crowding, encapsulation, and reaction volumes all profoundly affect stochastic variation of gene expression, which in turn impacts the choice between mass-action and ordinary differential equations for prediction of protein synthesis. In addition, cell-free systems lack a continuous supply of substrates, supplementary TFs, and chaperones, which could dramatically alter the rates of peptide and/or metabolite production *in vivo*. These factors could change kinetic parameters that *in vitro* systems operate within. There are other cellular processes, such as self-repair (Witkin, 1976; Demple and Halbrook, 1983; Demple and Harrison, 1994; Aas et al., 2003) and proofreading (Brutlag and Kornberg, 1972; Cline et al., 1996) that have not been considered when constructing cell-free synthetic systems.

While these missing features of *in vitro* systems could make it non-trivial to adapt existing computational tools for the design of cell-free systems, the minimality of cell-free systems provides a unique research opportunity to understand functioning of cells from a bottom-up perspective. We have detailed several projects that quantify the effects of molecular phenomena such as encapsulation, molecular crowding, and reaction volumes on the performance of *in vitro* transcription and translation. These projects could provide insights into key molecular phenomena that impact gene expression *in vivo*. Models that detail the impact of energy supply and molecular building blocks on protein synthesis in cell-free systems could similarly demonstrate how molecular transporters and secondary metabolic reactions modulate homeostasis of natural cells. Exploratory models for *in vitro* pathways could considerably speed the assembly of cell-free circuits, and provide excellent platforms for testing hypotheses of how complex processes, such as self-repair and proofreading, influence dynamical behavior of synthetic circuits. These automatic *in vitro* network assemblers could also form the fundamental tools for creating an integrated model of artificial cells.

Artificial cells represent unique *in vitro* platforms for studying fundamental principles of biochemical pathways. Indeed, they have been used to measure differences in the expression and stochastic variation of gene circuits caused by encapsulation. To create predictive models of artificial cells, existing design tools of gene circuits could be integrated with models of liposomes. Such whole-artificial-cell models could be used to predict the response of artificial cells to osmotic pressure and to understand plausible co-regulation of system dynamics by membranes and gene circuits.

The computational tools consolidated in this review establish a foundation for mathematical comparison between *in vivo* and *in vitro* biological phenomena. Computer simulation allows researchers to accelerate the pace of scientific inquiry and build a common framework for designing biological networks. *In vitro* reactions remain a powerful tool for experimental biologists, and as the field of biology becomes increasingly quantitative, it is important to take advantage of the flexibility of cell-free systems to test model predictions under simplified and minimal conditions. We envision that studies of cell-free and *in vivo* synthetic systems will reveal cryptic non-genetic factors, network structures, and spatial organization of cellular components that may modulate robustness of synthetic biological systems.

## Conflict of Interest Statement

The authors declare that the research was conducted in the absence of any commercial or financial relationships that could be construed as a potential conflict of interest.
